# Pan-caspase inhibitors induce secretion of HIV-1 latency reversal agent lymphotoxin-alpha from cytokine-primed NK cells

**DOI:** 10.1038/s41420-025-02330-1

**Published:** 2025-02-04

**Authors:** Zamaneh Hajikhezri, Ioannis Zygouras, Anders Sönnerborg, Robert van Domselaar

**Affiliations:** 1 Division of Infectious Diseases, ANA Futura Laboratory, Department of Medicine Huddinge, Karolinska Institutet, Stockholm, Sweden; 2 Department of Clinical Microbiology, Karolinska University Hospital Huddinge, Stockholm, Sweden

**Keywords:** NK cells, HIV infections

## Abstract

The persistence of HIV-1 latency reservoirs in CD4^+^ T cells is a significant obstacle for curing HIV-1. Shock-and-kill strategies, which aim to reactivate latent HIV-1 followed by cytotoxic clearance, have shown limited success in vivo due to insufficient efficacy of latency reversal agents (LRAs) and off-target effects. Natural killer (NK) cells, with their ability to mediate cytotoxicity independent of antigen specificity, offer a promising avenue for enhancing the shock-and-kill approach. Previously, we observed that pan-caspase inhibitors induce NK cells to secrete an LRA in vitro. Here, we aimed to identify this LRA using a targeted proteomic approach. We identified lymphotoxin-α (LTα) as the key LRA secreted by NK cells following pan-caspase inhibitor treatment. LTα was shown to significantly induce HIV-1 LTR promoter activity, a hallmark of viral reactivation. Neutralization of LTα effectively abolished the observed LRA activity, confirming its central role. Moreover, cytokine-primed but not resting human primary NK cells exhibited LRA activity that could be neutralized with LTα neutralizing antibodies. Finally, pan-caspase inhibitor treatment did not decrease the ability of the cytokine-primed NK cells to kill target cells. These findings demonstrate that cytokine-primed NK cells, through LTα secretion, can effectively reactivate latent HIV-1 following pan-caspase inhibitor treatment, without compromising NK cell cytotoxicity. This highlights a potential enhancement strategy utilizing NK cells for shock-and-kill approaches in HIV-1 cure research.

## Introduction

The formation of HIV-1 latency reservoirs in mostly memory CD4^+^ T cells is a major barrier for a functional cure of HIV-1 infection. Although antiretroviral therapy (ART) can successfully block HIV-1 replication resulting in undetectable blood plasma viral loads in patients and preventing disease progression, viremia rapidly reappears from these latency reservoirs when ART is stopped [1, 2]. One of the potential strategies to eradicate the latency reservoirs is the shock-and-kill strategy, which aims to reactivate HIV-1 from the latently infected cells using latency reversal agents (LRAs) followed by elimination through cytotoxic lymphocytes, e.g. HIV-1-specific CD8^+^ T cells and natural killer (NK) cells [3, 4]. As of yet, LRAs have not resulted in a desired reduction in viral reservoir in vivo due to low efficacy and off-target effects in predominantly other immune cells [5–8]. Therefore, combining multiple LRAs with synergistic effects or including LRAs in cellular immunotherapies could be a way forward for a successful shock-and-kill strategy. Cellular immunotherapies focus on harnessing immune cells to eradicate tumor cells or virus-infected cells. Both T cell-based and NK cell-based therapies have emerged as promising strategies in the treatment of cancers, but also for the treatment of virus infections like HIV-1 [9–12]. However, T cell-based therapies have as a disadvantage that target cells can escape antigen recognition and have been associated with cytokine release syndrome. NK cells do not rely on antigen-driven activation. Instead, NK cell-mediated cytotoxicity is determined by the balance of inhibitory and activating receptor signaling. And besides their ability to kill cells, NK cells also aid in shaping and maintaining adaptive immune responses [11, 13].

NK cell-based strategies could be utilized to eliminate HIV-1 latent reservoirs when combined with a shock-and-kill strategy. Recently, it was shown that human peripheral blood NK cells together with an LRA can efficiently delay HIV-1 rebound from latency following ART interruption in vivo in a humanized mouse model [14]. This was accompanied by a reduction in the diversity of viral clones, thus diminishing the HIV-1 latency reservoir [14]. In another study, primary HIV-1 latently infected CD4^+^ T cells were stimulated with LRAs and then co-cultured with both autologous NK cells and bi-specific antibodies that bind CD16 on NK cells and the HIV-1 envelope protein Env on reactivated HIV-1-infected cells [15]. This resulted in enhanced NK cell-mediated killing of the HIV-1-infected cells and increased reduction of the HIV-1 latency reservoir [15]. We have shown that pan-caspase inhibitors modulate NK cell activity resulting in the release of an unidentified LRA that synergizes with other LRAs such as BET bromodomain inhibitor JQ1 or protein kinase C agonist prostratin [16].

In the present study, we used a targeted proteomic approach to identify lymphotoxin-α (LTα) as the secreted LRA from pan-caspase inhibitor-treated NK cells. In addition, we showed that cytokine stimulation is a prerequisite for human primary NK cells to secrete LTα after pan-caspase inhibitor treatment and that this treatment does not compromise the cytotoxic potential of these NK cells.

## Results

### Changes in secretome of pan-caspase inhibitor treated NK cells

**Fig. 1:**
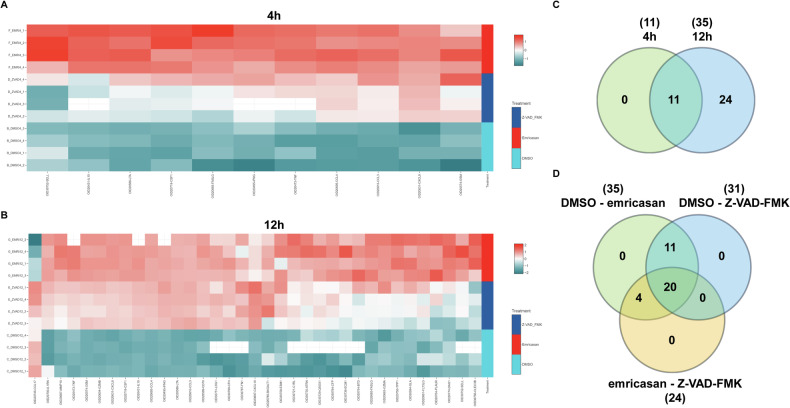
Secretome analysis of pan-caspase inhibitor-treated NK cells. NK lymphoma cell line KHYG-1 was incubated with DMSO (0.5%), Z-VAD-FMK (50 µM), or emricasan (50 µM) for 4 h or 12 h (each condition *n* = 4) after which supernatants were collected and relative protein levels were analyzed by PEA Olink panels Inflammation I and Inflammation II. Heatmaps of all secreted proteins with significantly different protein levels among the three groups at 4 h (**A**) and 12 h (**B**). Venn diagrams depicting the number of secreted proteins with significantly different protein levels between the 4 h and 12 h time points (**C**), and between the three groups at 12 h (**D**).

**Fig. 2:**
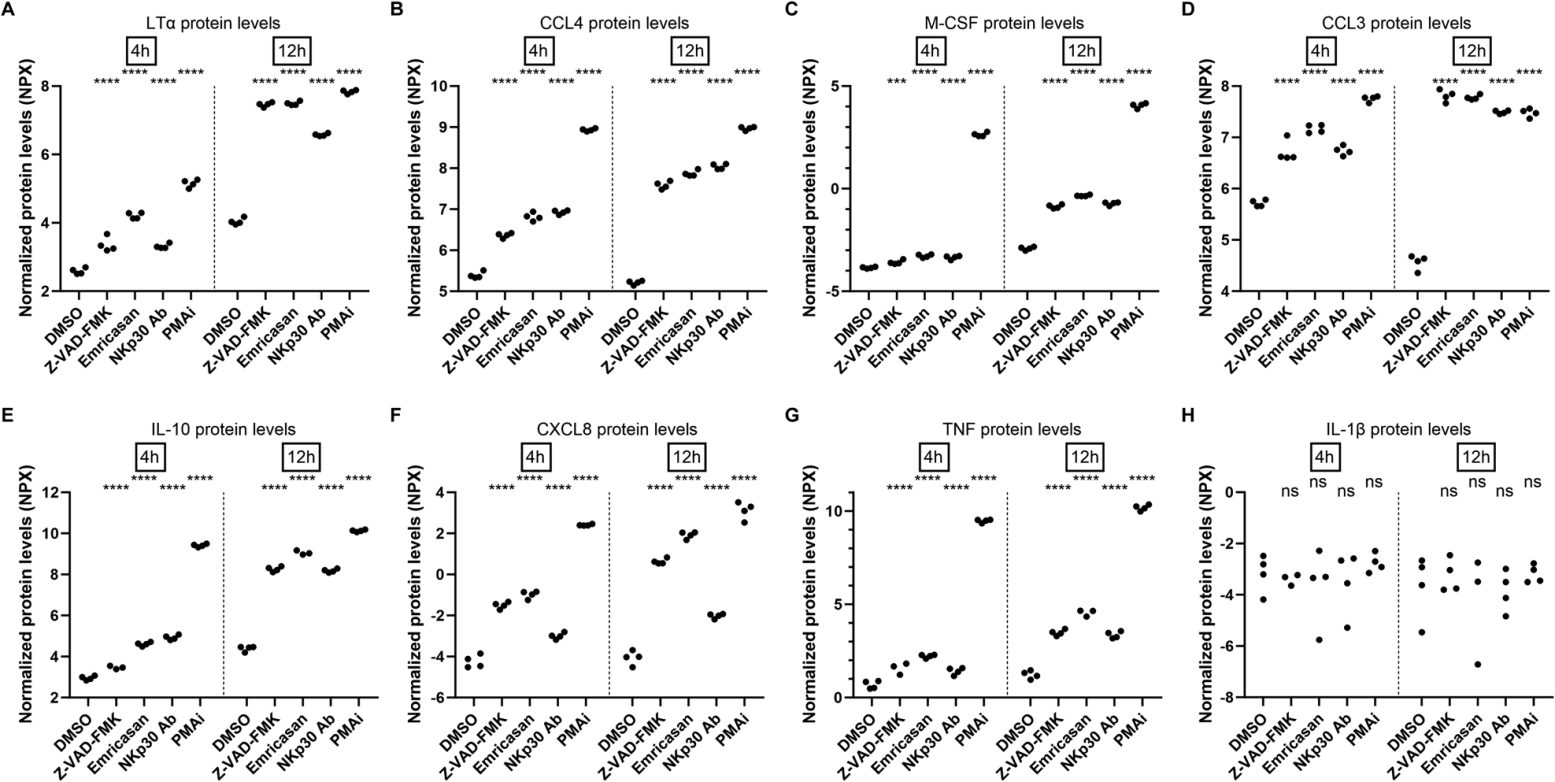
Innate cytokines are secreted by NK cells upon pan-caspase inhibitor-treatment. Normalized protein levels (NPX) (*n* = 4) for **A** LTα, **B** CCL4, **C** M-CSF, **D** CCL3, **E** IL-10, **F** CXCL8, and **G** TNF are depicted. **H** Data for IL-1β is shown as an example of a protein that showed no difference in protein level between treatments. Two-way ANOVA was performed with comparisons for each analyte and for each time point to the DMSO control. (ns, non-significant: ***p < 0.001, ****p < 0.0001).

To identify the LRA that is secreted by NK cells upon pan-caspase inhibitor treatment, we employed a targeted proteomic approach to screen for secreted proteins in the supernatants of NK cells. We incubated NK lymphoma cell line KHYG-1 with the pan-caspase inhibitors Z-VAD-FMK or emricasan and collected supernatants after 4 h or 12 h. Since we also wanted to assess to what extent the pan-caspase inhibitors could activate NK cells, we included NK cells stimulated with a strong activator, namely PMA and ionomycin (PMAi) and a more physiological mild activator using an agonist antibody against NK cell activating receptor NKp30. We first verified whether the supernatants contain LRA activity using a reporter cell line in a luciferase assay. In this assay, we can assess whether supernatants can induce HIV-1 LTR promotor activity as we showed before [16]. Supernatants of pan-caspase inhibitor treated NK cells and NKp30 activated NK cells showed increased LRA activity from 4 h to 12 h treatment (Supplementary Fig. 1). PMAi itself already activated LTR promotor activity in the reporter cells and thus supernatants of PMAi-treated NK cells could not be properly assessed for LRA activity in our system. Then, these supernatants were analyzed for the relative protein levels of 733 proteins using a PEA platform. As expected, PMAi dramatically changed the secretome profile already after 4 h of treatment as well as after 12 h (Supplementary Fig. 2). Both pan-caspase-inhibitors showed similar secretome profiles as the NKp30 activator (Supplementary Fig. 2). When comparing the pan-caspase inhibitor-treated cells to control cells, we observed few changes in the secretome profile after 4 h of treatment with only 11 statistically different protein levels (Fig. 1A and Table 1). After 12 h of treatment, we observed more changes in the secretome with 35 proteins being statistically different in protein levels (Fig. 1B–D and Table 2). Among these 35 proteins were CCL3, CCL4, and IL-10, which we have previously reported to be secreted by pan-caspase inhibitor-treated NK cells (Table 2 and Fig. 2) [16]. Other proteins that showed increased levels were cytokines such as LTα, M-CSF/CSF1, IL-8/CXCL8 and TNF (Table 2 and Fig. 2).

**Table 1:** List of significantly different protein levels between the three treatment groups (DMSO; Z-VAD-FMK; emricasan) at the 4 h time point.

Assay	UniProt	Olink® panel	*p* value	Adjusted *p* value
CCL4	P13236	Inflammation	3.940e-9	0.000002888
CXCL8	P10145	Inflammation	1.795e-8	0.000004946
IL10	P22301	Inflammation	2.024e-8	0.000004946
CCL3	P10147	Inflammation	3.385e-7	0.00004962
LTA	P01374	Inflammation	2.719e-7	0.00004962
CSF1	P09603	Inflammation	0.000009067	0.001108
TNF	P01375	Inflammation	0.00002320	0.002430
FASLG	P48023	Inflammation	0.0001060	0.009713
IFNG	P01579	Inflammation	0.0001485	0.01210
OSM	P13725	Inflammation	0.0003820	0.02800
SELL	P14151	Inflammation_II	0.0005145	0.03428

**Table 2:** List of significantly different protein levels between the three treatment groups (DMSO; Z-VAD-FMK; emricasan) at the 12 h time point.

Assay	UniProt	Olink® panel	*p* value	Adjusted *p* value
LTA	P01374	Inflammation	2.617e-13	1.918e-10
CCL4	P13236	Inflammation	3.418e-12	1.253e-9
CSF1	P09603	Inflammation	8.079e-12	1.974e-9
CCL3	P10147	Inflammation	1.370e-11	2.511e-9
IL10	P22301	Inflammation	3.903e-11	5.721e-9
CXCL8	P10145	Inflammation	1.242e-10	1.517e-8
GZMB	P10144	Inflammation	4.708e-10	4.469e-8
IFNG	P01579	Inflammation	4.990e-10	4.469e-8
OSM	P13725	Inflammation	5.488e-10	4.469e-8
CXCL10	P02778	Inflammation	6.108e-9	4.477e-7
TNF	P01375	Inflammation	3.292e-8	0.000002194
CD70	P32970	Inflammation	1.656e-7	0.00001011
MMP10	P09238	Inflammation	1.892e-7	0.00001067
LRG1	P02750	Inflammation_II	0.000002003	0.0001049
FASLG	P48023	Inflammation	0.000002423	0.0001110
TPP1	O14773	Inflammation	0.000002423	0.0001110
CFH	P08603	Inflammation_II	0.00001315	0.0005669
C1RL	Q9NZP8	Inflammation_II	0.00002750	0.001120
DAG1	Q14118	Inflammation	0.00003075	0.001127
GLA	P06280	Inflammation_II	0.00002947	0.001127
CFP	P27918	Inflammation_II	0.00004246	0.001482
FN1	P02751	Inflammation_II	0.00004872	0.001623
SELL	P14151	Inflammation_II	0.00007005	0.002232
QSOX1	O00391	Inflammation_II	0.00009513	0.002905
GZMA	P12544	Inflammation	0.00009956	0.002919
FLT3LG	P49771	Inflammation	0.0001490	0.004202
BTD	P43251	Inflammation_II	0.0001862	0.005025
CLEC3B	P05452	Inflammation_II	0.0001919	0.005025
IL1RN	P18510	Inflammation	0.0003240	0.008190
PLAUR	Q03405	Inflammation	0.0004770	0.01165
ATRN	O75882-2	Inflammation_II	0.0005053	0.01195
ESM1	Q9NQ30	Inflammation	0.0006671	0.01528
ECM1	Q16610	Inflammation_II	0.001000	0.02222
CCL17	Q92583	Inflammation	0.001105	0.02381
B4GALT1	P15291	Inflammation	0.002159	0.04522

### Lymphotoxin-α is responsible for the LRA activity in supernatants of NK upon pan-caspase inhibitor treatment

**Fig. 3:**
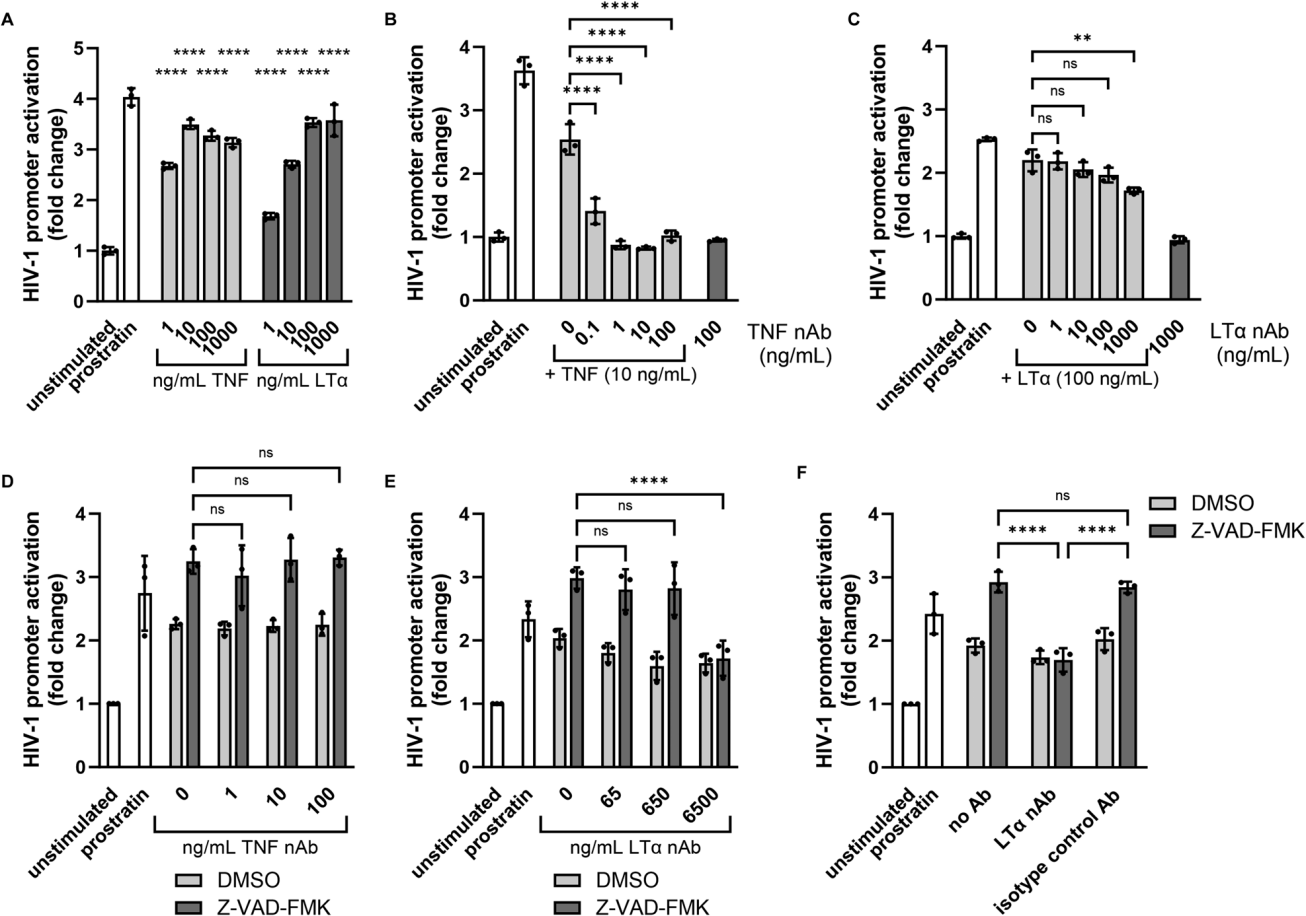
Secreted LTα but not TNF induces HIV-1 promoter reactivation. **A** TZM-bl cells were treated with different concentrations of TNF or LTα overnight. Activation of the HIV-1 LTR promoter was assessed by subjecting these cells to a luciferase assay. Data points are plotted as mean ± SD from technical triplicates. Two-way ANOVA was performed with comparisons for each cytokine to the unstimulated control. **B**, **C** Optimal TNF and LTα concentrations were pre-incubated with various concentrations of the corresponding neutralizing Ab (nAb) against either TNF or LTα. To determine the neutralizing capacity of the nAb, these mixtures were added to TZM-bl cells overnight followed by a luciferase assay (gray bars). TZM-bl cells incubated with only nAb was used as a control (black bars). Data points are plotted as mean ± SD from technical triplicates. Ordinary one-way ANOVA was performed on the depicted comparisons. **D**, **E** KHYG-1 cells were incubated with DMSO (0.5%) or Z-VAD-FMK (50 µM) overnight. Supernatants were collected and pre-incubated with concentration ranges of either TNF or LTα nAb before adding to TZM-bl cells overnight followed by a luciferase assay. Data points are plotted as mean ± SD from three individual experiments each with technical triplicates. Two-way ANOVA was performed with comparisons to 0 ng/mL nAb for each treatment. Only comparisons for Z-VAD-FMK treatment are shown. All comparisons for DMSO treatment were non-significant. **F** KHYG-1 cells were incubated with DMSO (0.5%) or Z-VAD-FMK (50 µM) overnight. Supernatants were collected and pre-incubated with no antibody, LTα nAb (6.5 µg/mL), or IgG2A isotype control (6.5 µg/mL) before adding to TZM-bl cells overnight followed by a luciferase assay. Data points are plotted as mean ± SD from three individual experiments each with technical triplicates. Two-way ANOVA was performed. Only comparisons for Z-VAD-FMK treatment are shown. All comparisons for DMSO treatment were non-significant. (ns, non-significant: **p < 0.01, ****p < 0.0001).

From all identified secreted proteins, TNF and LTα are known to induce HIV-1 reactivation. Since the PEA assay does not provide absolute quantification of the analyzed proteins, we further examined whether any of these two cytokines were responsible for the LRA activity in pan-caspase inhibitor-treated NK cell supernatants. First, we tested whether TNF and LTα indeed activated the HIV-1 LTR promotor in our luciferase assay model. TNF activated the HIV-1 promoter at a more than 10-fold lower concentration than LTα (Fig. 3A). Next, we analyzed the optimal concentration of both TNF and LTα nAbs to abolish the LRA activity of the two cytokines (Fig. 3B, C). In contrast to TNF and TNF nAb, neutralization of an LRA-inducing concentration of LTα required high concentrations of LTα nAb. Therefore, we had to add high concentrations of the LTα nAb in the following experiments. Finally, we incubated KHYG-1 cell supernatants with the nAbs before measuring LRA activity. TNF nAb failed to inhibit the LRA activity of Z-VAD-FMK-treated KHYG-1 supernatants (Fig. 3D). However, LTα nAb could completely abolish the LRA activity of Z-VAD-FMK-treated KHYG-1 supernatants (Fig. 3E), whereas an isotype control antibody did not (Fig. 3F). This strongly indicates that it was LTα secreted from NK cells after pan-caspase inhibitor treatment that induced HIV-1 reactivation.

### Cytokine-priming of human primary NK cells is required for Z-VAD-FMK to induce LRA activity

**Fig. 4:**
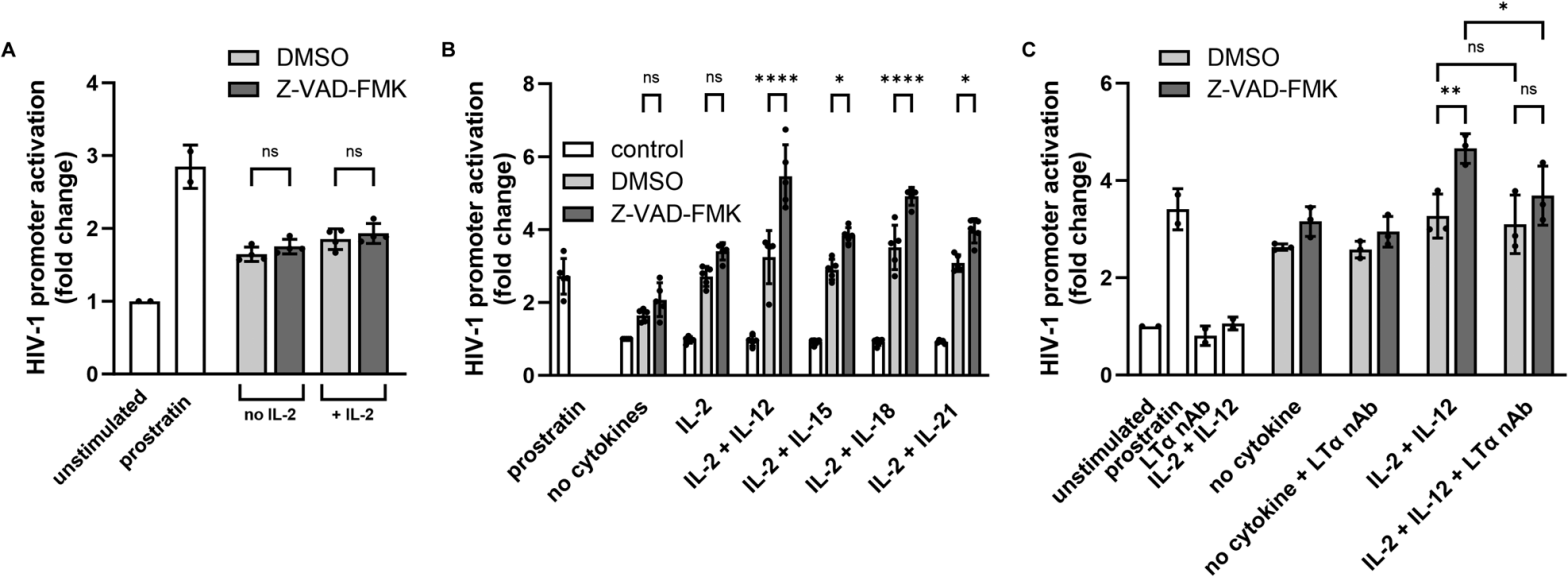
Cytokine-primed human primary NK cells induce latency reversal through LTα. **A** Primary NK cells (from 4 donors in 2 independent experiments) were cultured with no or low dose of IL-2 for 24 h and then treated with Z-VAD-FMK (50 µM) or without (DMSO, 0.5%) for another 24 h after which supernatants were used on reporter cells TZM-bl that were subjected to luciferase assay (technical triplicates for each donor). Data points are plotted as mean ± SD from all donors (*n* = 4). Two-way ANOVA was performed on the depicted comparisons. **B** Primary NK cells (from 5 donors, each as independent experiment) were cultured with various cytokine combinations for 24 h and then treated with Z-VAD-FMK (50 µM) or without (DMSO, 0.5%) for another 24 h after which supernatants were used on reporter cells TZM-bl that were subjected to luciferase assay (technical triplicates for each donor). Cytokine controls are TZM-bl cells cultured with the respective cytokines. Data points are plotted as mean ± SD from all donors (n = 5). Two-way ANOVA was performed on the depicted comparisons. **C** Primary NK cells (from 3 donors, across two independent experiments) were cultured with various cytokine combinations for 24 h and then treated with Z-VAD-FMK (50 µM) or without (DMSO, 0.5%) for another 24 h after which supernatants were collected. Supernatants were pre-incubated with LTα nAb (6.5 µg/mL) before adding to reporter cells TZM-bl that were subjected to luciferase assay (technical triplicates for each donor). Data points are plotted as mean ± SD from all donors (*n* = 3). Two-way ANOVA was performed on the depicted comparisons. (ns, non-significant: *p < 0.05, **p < 0.01, ****p < 0.0001).

Previously, we have shown that Z-VAD-FMK could induce LRA activity from human primary NK cells primed with IL-21 and a high dose of IL-2 [16]. Now, we examined whether cytokine priming of human primary NK cells was required for Z-VAD-FMK to exert its indirect LRA effect. First, we cultured enriched human primary NK cells without any cytokines (resting NK cells) or a low dose of IL-2. Z-VAD-FMK failed to induce LRA activity from NK cells under these two conditions (Fig. 4A). However, when we combined a low dose of IL-2 with either IL-12, IL-15, IL-18, or IL-21, we detected increased LRA activity in the supernatant after Z-VAD-FMK-treated human primary NK cells, particularly with the IL-2/IL-12 and IL-2/IL-18 cytokine combinations (Fig. 4B). LTα nAb also abolished the LRA activity in supernatants of IL-2/IL-12-primed Z-VAD-FMK-treated human primary NK cells (Fig. 4C). This indicates that NK cells require cytokine-induced signaling for the pan-caspase inhibitors to secrete LTα and exert their HIV-1 latency reversal effect.

**Fig. 5:**
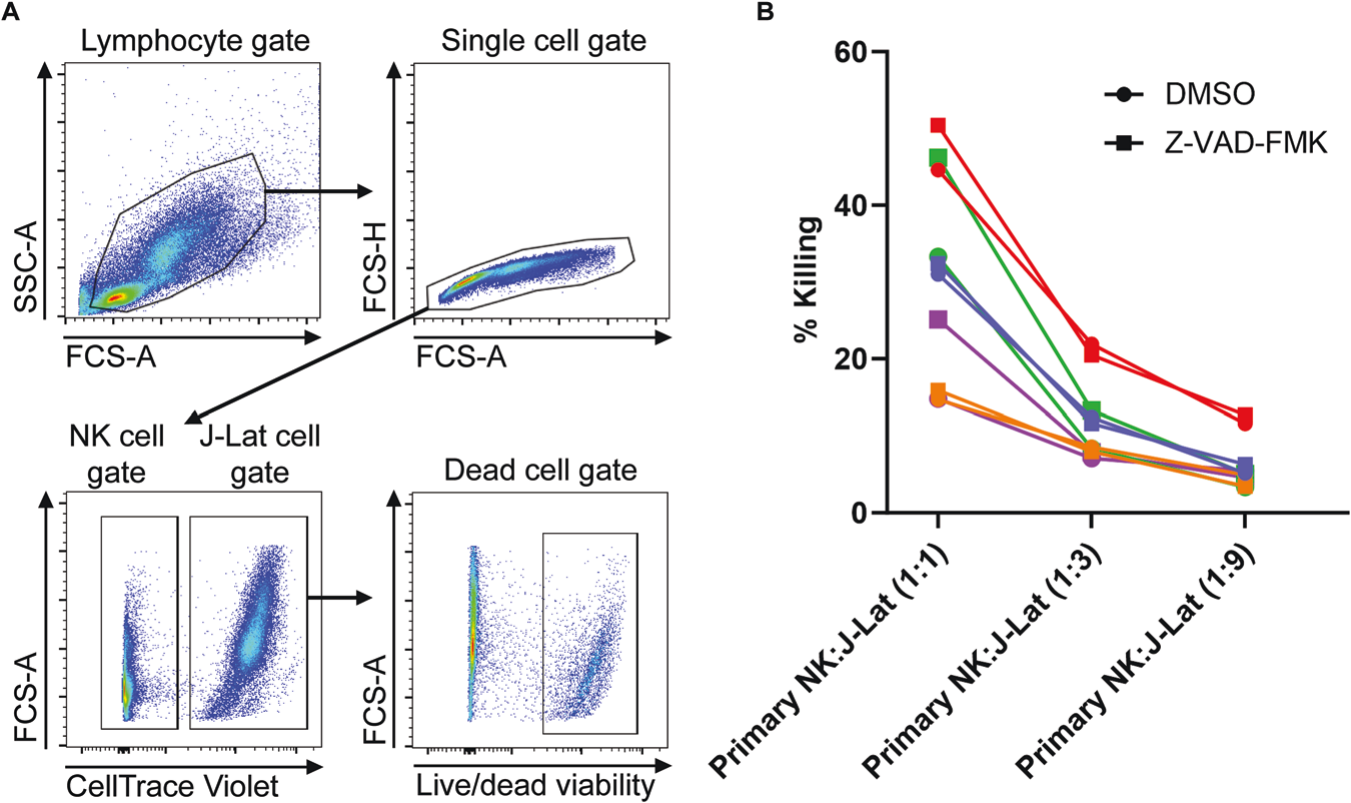
Pan-caspase inhibitor treatment does not compromise NK cell cytotoxic potential. Human primary NK cells (from 5 donors) were primed with IL-2/IL-12 followed by Z-VAD-FMK or DMSO incubation for 1 h and co-cultured with J-Lat target cells at various effector-to-target (*E*:*T*) ratios (1:1, 3:1, and 9:1) for 4 h. NK cell cytotoxicity was assessed using flow cytometry to determine the percentage of target cell death. **A** Flow cytometry gating strategy for the calculation of NK cell-mediated target cell killing. **B** Data points are plotted for each individual donor (*n* = 5) where each color represents an individual donor. The shapes represent different treatments: circles (DMSO) and squares (Z-VAD-FMK). Two-way RM ANOVA showed a significant difference (**p < 0.01) when comparing the matched DMSO treatment values against Z-VAD-FMK treatment values at the 1:1 *E*:*T* ratio. For the 1:3 and 1:9 *E*:*T* ratios it was non-significant.

Since we are evaluating LRA secretion in the context of a shock-and-kill strategy, the pan-caspase inhibitor should not compromise the cytotoxic potential of the NK cells. To assess this, we pre-incubated IL-2/IL-12-primed human primary NK cells with Z-VAD-FMK before performing a co-culture with J-Lat cells as target cells at various *E:T* ratios for 4 h followed by flow cytometry analysis (Fig. 5A). For each donor, we did not observe any decrease in the percentage of target cell death mediated by Z-VAD-FMK-treated NK cells compared to control NK cells (Fig. 5B). For the 1:1 *E*:*T* ratio there was even a slight increase in cytotoxic potential, but not at lower *E*:*T* ratios. Altogether, this suggests that pan-caspase inhibition within cytokine-primed human primary NK cells can mediate HIV-1 reactivation through release of LTα without negatively affecting NK cell cytotoxic potential.

## Discussion

NK cell-based therapies are currently being explored in the search for a functional cure for HIV-1 infection. NK cells lack antigen specificity, but instead identify a wide variety of danger and stress signals allowing them to quickly adapt to the environment and to act accordingly. This opens possibilities to modulate NK cell activity for various therapeutic approaches. We explored the effects of pan-caspase inhibitor treatment on NK cells regarding induced LRA activity and killing potential. The pan-caspase inhibitors induced secretion of a variety of cytokines, but far less than the strong activators PMA and ionomycin. However, not all proteins listed were increased to a biological relevant level. For example, despite that the strong LRA TNF was significantly increased in the supernatant after pan-caspase inhibitor treatment, it was not responsible for our observed NK cell-mediated LRA activity. Thus, our proteomic approach using PEA is very sensitive in identifying small increases in protein levels, but thereby also potentially overestimates the number of increased biologically relevant proteins in our context. However, it did help us to identify LTα, a member of the TNF superfamily, as the responsible LRA secreted by NK cells after pan-caspase inhibitor treatment.

Although LTα, previously known as TNFβ, was identified as an LRA decades ago [17, 18], it has been largely underappreciated because of its weaker potency to induce HIV-1 reactivation compared to TNF, previously known as TNFα. Unlike other members of the TNF superfamily, LTα is secreted as a soluble homotrimer but can also exist as a heterotrimer with membrane-bound LTβ, mostly as LTα1β2, on the cell surface. Since the LTβ-receptor is not expressed by T cells [19], and our observed LRA activity was identified in the supernatants of pan-caspase inhibitor-treated NK cells that was lost after LTα neutralization, our data strongly indicates that the soluble LTα homotrimer is responsible for HIV-1 reactivation. Like TNF, LTα interacts with receptors TNFR1 and TNFR2 to induce canonical or non-canonical NF-κB signaling [20, 21], respectively, which likely in turn induces HIV-1 reactivation [22]. For a potential shock-and-kill strategy including NK cells, secreted soluble LTα could reach many cells non-specifically, thereby reaching HIV-1 latently infected cells. Then, after HIV-1 reactivation, the NK cells can specifically target and kill the HIV-1 reactivated cells through cell-cell contact.

The limited cytokine release of pan-caspase inhibitor-treated NK cells might result in reduced complications in vivo, such as cytokine release syndrome, when used in a therapeutic strategy. Although we have not studied the effect of pan-caspase inhibitors on other immune cells, the pan-caspase inhibitor emricasan has been used in clinical trials for other clinical purposes and was found to be safe and tolerable [23–29]. Additionally, the pan-caspase inhibitors appeared to be ineffective on resting NK cells and only worked on cytokine-primed NK cells without compromising their cytotoxic potential. NK cells can respond to several cytokines that cause NK cells to proliferate, secrete other cytokines, and enhance cytotoxic potential. Currently, strategies are being investigated to enhance NK cell function with cytokine treatments [11, 13]. Thus, for a potential therapeutic shock-and-kill strategy, cytokine treatment and pan-caspase inhibitor treatment could be combined to make the NK cells susceptible for the pan-caspase inhibitor shock effect and to enhance the killing. Furthermore, we have previously shown that there is synergy with other LRAs such as BET bromodomain inhibitor JQ1 and protein kinase C agonist prostratin [16]. Combining multiple LRAs could overcome therapeutic barriers such as drug toxicity and low drug efficacy.

Pan-caspase inhibitors might have a negative effect on NK cells as caspases might play underappreciated non-canonical roles in lymphocytes. Active caspase-8, which is essential for death receptor-induced apoptosis, is required for activation-induced proliferation of T cells [30]. TCR-mediated T cell activation requires caspase-8 to activate NF-κB signaling, and Z-VAD-FMK blocks this NF-κB signaling [31]. TCR-mediated NF-κB activation is known for HIV-1 reactivation and thus pan-caspase inhibitors would block this HIV-1 reactivation pathway. However, previously, we have shown that Z-VAD-FMK does not negatively impact the mechanism by which Z-VAD-FMK-treated NK cells reactivate HIV-1 within T cells [16]. In regards to NK cells, active caspase-8 has been shown to be required for IL-2-induced proliferation of NK cells as well as release of IFN-γ and TNF from activated NK cells that can be inhibited by pan-caspase inhibitor Z-VAD-FMK [32]. Finally, caspase-8 deficient NK cells fail to activate NF-κB signaling after CD16/Fc receptor stimulation [31]. This could suggest that pan-caspase inhibitors could block or redirect the intracellular NF-κB signaling within lymphocytes, thereby modulating the activation status of these cells. Since resting NK cells are unaffected by pan-caspase inhibitor treatment, at least in regard to LRA activity, cytokine priming might induce temporary caspase activity that allows the inhibitor to irreversible bind and change the functional outcome of NK cells. However, this requires a better understanding of how caspases are involved in the activation signaling pathways of NK cells during or after cytokine-priming. Also, more specific drugs can be developed or repurposed to modulate NK cell activity without blocking the entire caspase-mediated apoptosis signaling pathway within the target cells that would compromise the essential killing of the HIV-1 reactivated cells in a shock-and-kill strategy. Finally, a functional cure of HIV-1 infection will require a multi-faceted strategy in which NK cells could play crucial roles. Antibody-based approaches, such as broadly neutralizing antibodies, can induce NK cell-mediated antibody-mediated cellular cytotoxicity [15, 33–35]. Shock-and-kill approaches with LRAs require cytotoxic lymphocyte-mediated killing of the reactivated cells [3, 4]. Therefore, (pan-)caspase inhibitor treatment of NK cells could potentially be an enhancement approach in a shock-and-kill strategy that could be combined with antibody-based approaches for a functional HIV-1 cure.

## Materials/Subjects and methods

### Cell culture

Cells were cultured in 5% CO_2_ at 37 °C. TZM-bl cells were maintained in Dulbecco’s modified Eagle medium (DMEM, Gibco/ThermoFisher Scientific, Waltham, MA, USA) supplemented with 10% fetal bovine serum (FBS, Sigma/Merck, Darmstadt, Germany), 2 mM L-glutamine (Sigma), 0.1 mM MEM Non-Essential Amino Acids (Gibco), and 20 U/mL penicillin combined with 20 µg/mL streptomycin (Sigma). KHYG-1 cells (#ACC 725, DSMZ, Braunschweig, Germany) were maintained in Roswell Park Memorial Institute 1640 medium with GlutaMAX (RPMI, Gibco) supplemented with 10% FBS, 25 mM HEPES, 20 U/mL penicillin and 20 μg/mL streptomycin, and 100 U/mL of recombinant human interleukin-2 (IL-2, PeproTech/ThermoFisher Scientific). J-Lat 10.6 cells were maintained in RPMI medium supplemented with 10% FBS, 25 mM HEPES, 20 U/mL penicillin and 20 μg/mL streptomycin.

### Enrichment of primary NK cells

Peripheral blood mononuclear cells (PBMCs) were isolated from buffy coats of anonymous blood donors using Ficoll density centrifugation (Ficoll® Paque Plus, Sigma) and SepMate™-50 tubes (StemCell Technologies, Vancouver, Canada), aliquoted and stored in liquid nitrogen. Frozen aliquots of PBMCs were thawed and rested overnight in AIM-V medium (Gibco) supplemented with 5% human AB serum (Sigma) at a cell density of 10 × 10^6^ cells/mL. The following day, NK cells were negatively selected using the EasySep™ Human NK Cell Enrichment Kit (StemCell Technologies) according to the manufacturer’s protocol. Enriched NK cells were kept in AIM-V medium supplemented with 5% human AB serum and indicated cytokines at a cell density of 1 × 10^6^ cells/mL overnight. Then, these cells were used for luciferase or NK cell killing assays.

### Reagents & antibodies

Pan-caspase inhibitors Z-VAD-FMK and emricasan were purchased from Enzo Life Sciences (Farmingdale, NY, USA) and Selleckchem (Houston, TX, USA), respectively. Recombinant human IL-12 p70, IL-15, IL-21, TNF and LTα were purchased from PeproTech and recombinant human IL-18 from ThermoFisher Scientific. Human TNF neutralizing rabbit monoclonal antibody (clone D1B4, cat. no. 7321) was purchased from Cell Signaling Technologies (Danvers, MA, USA). Human LTα neutralizing mouse monoclonal antibody (clone 5807R, cat. no. MAB621R), mouse IgG2a isotype control (clone 133304, cat. no. MAB0031), and human NKp30 agonistic mouse monoclonal antibody (clone 210847, cat. no. MAB18491) were purchased from R&D Systems (Minneapolis, MN, USA). Prostratin, phorbol 12-myristate 13-acetate (PMA), and ionomycin were purchased from Sigma. CellTrace™ Violet proliferation kit and LIVE/DEAD Fixable Near IR Dead Cell Stain Kit were obtained from ThermoFisher Scientific.

### Proteomic profiling using proximity extension assay

KHYG-1 (1 × 10^6^/mL) were seeded in complete RPMI with 100 U/mL IL-2 in 40 wells of 12-well plates. Eight wells each were incubated with any of the 5 following treatments: (1) 0.5% DMSO; (2) 50 µM Z-VAD-FMK; (3) 50 µM emricasan; (4) 1 µg/mL agonistic NKp30 antibody; (5) 10 ng/mL PMA and 1 µg/mL ionomycin. Additionally, complete medium without IL-2 was added to 4 wells, which represented blanks for the proximity extension assay (PEA). Supernatants from 4 wells were collected after 4 h and the other 4 wells after 12 h of treatment incubation. Part of the supernatants were subjected to a luciferase assay to verify LRA activity. For proteomic profiling, 80 µL supernatant of all 44 conditions were randomized in half a 96-well plate and stored at −80 °C until sent for analysis. The relative protein abundance of 733 proteins from the Olink® Explore 384 Inflammation and Olink® Explore 384 Inflammation II were assessed using the PEA platform (Olink, Uppsala, Sweden). Data for relative protein abundance is given as normalized protein expression (NPX). ANOVA F-tests, post-hoc analysis and heatmap generations were performed with the online available Olink Statistical Analysis app (Olink). Assay samples with QC warnings were excluded from the analysis. Venn diagrams were created manually.

### Luciferase assay

TZM-bl cells (2 × 10^4^ cells) were seeded in 96-well plate one day prior to start of the experiment. NK cells (1 × 10^6^/mL) were incubated with Z-VAD-FMK (50 µM) or DMSO (0.5%) overnight. The next day, NK cell supernatants were collected. Then, medium from the TZM-bl cells was removed, and NK cell supernatants or media with indicated cytokines were added in triplicate (100–200 µL per well). For TNF and LTα neutralization, NK cell supernatants were first pre-incubated with the respective neutralizing antibody (nAb) or isotype control for 2 h at 37 °C. TZM-bl cells with only complete medium and RPMI with prostratin (6 µM) were used as controls. After one day of culture, supernatants were removed, washed once with PBS, and lysed in 50 µL Passive Lysis Buffer (Promega, Madison, WI, USA) for 30 min at 4 °C. Finally, 20 µL lysates were transferred into a white 96-well plate, 100 µL luciferase reagent (Promega) was added, and luminescence was measured on the Tecan Spark microplate reader (Tecan Group Ltd, Männedorf, Switzerland). For each experiment fold change was calculated by dividing the relative luminescence units (RLU) of each condition by the average RLU of TZM-bl cells cultured only with complete medium (unstimulated). TZM-bl cells incubated with LRA prostratin served as positive control.

### NK cell killing assay

J-Lat 10.6 cells were labeled with 2 µM CellTrace Violet dye one day prior to the start of co-culture. Enriched human primary NK cells from 5 different donors were cultured with IL-2 (50 U/mL) and IL-12 (50 ng/mL) for 24 h, followed by incubation with Z-VAD-FMK (50 µM) or DMSO (0.5%) for 1 h. Then, NK cells were washed twice, and added to J-Lat cells in various effector-to-target (*E*:*T*) ratios. After 4 h of co-culture, cells were collected, washed in ice-cold PBS and incubated with Near IR viability dye for 30 min at 4 °C in the dark. The cells were then washed with ice-cold PBS and fixed with 2% paraformaldehyde for 20 min at 4 °C in the dark. Then, cells were washed with ice-cold PBS one last time and finally resuspended in ice-cold PBS and stored at 4 °C until analysis. Flow analysis was performed on BD FACSVerse (BD Biosciences, Franklin Lakes, NJ, USA). The percentage of NK cell-mediated killing of target cells was calculated by determining the percentage of near IR viability dye-positive cells within the CellTrace Violet-positive cell population.

### Data analysis

Data was analyzed and plotted using GraphPad Prism v10.1.2 (San Diego, CA, USA). Statistical analysis using ordinary one-way or two-way ANOVA with multiple comparisons was performed using GraphPad Prism.

### Ethics statement

This study involved the use of buffy coats from individuals that donated blood at Karolinska University Hospital Huddinge. The participants provided their informed consent to participate in the research study. All samples were de-identified before receipt. We confirm that all methods were performed in accordance with institutional and national guidelines.

## Supplementary information

Supplementary information

Supplementary data set 1

## Data Availability

The full proteomic profiling data set is available online as Supplementary dataset 1.
