# Supplementary material for: Pan-caspase inhibitors induce secretion of HIV-1 latency reversal agent lymphotoxin-alpha from cytokine-primed NK cells
Source: Cell Death Discov. 2025 Feb 4;11:44. doi: 10.1038/s41420-025-02330-1 (PMC11794648; doi:10.1038/s41420-025-02330-1)
Supplement: Supplementary file 1 — Supplementary information [file 41420_2025_2330_MOESM1_ESM.pdf]

Supplementary figures

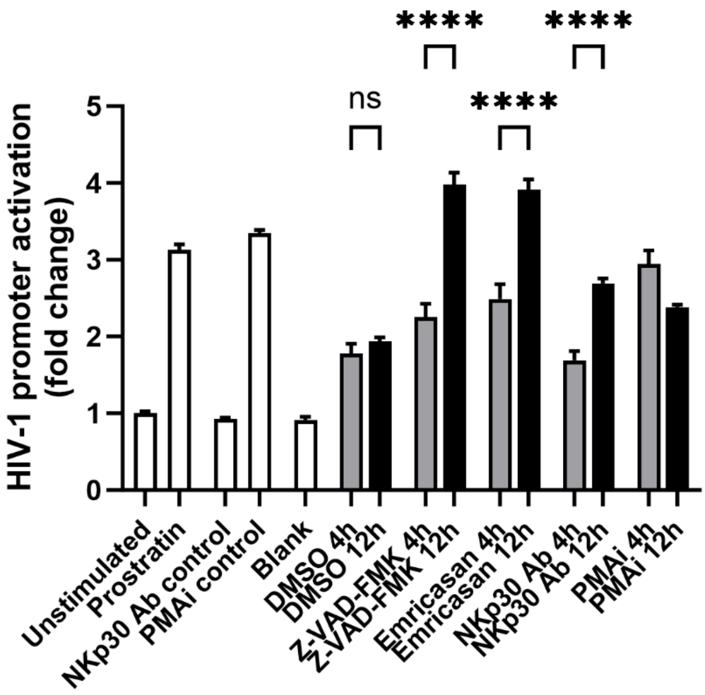

Supplementary figure 1. LRA activity in supernatants of NK cells after various treatments.

NK lymphoma cell line KHYG-1 was incubated with DMSO (0.5 %), Z-VAD-FMK (50 μM), emricasan (50 μM), agonistic NKp30 antibody (1 μg/mL), or PMAi (10 ng/mL PMA and 1 μg/mL ionomycin) for 4 h or 12 h (each condition n=4) after which supernatants were used on reporter cells TZM-bl that were subjected to luciferase assay. Data points are plotted as mean ±SD from the biological replicates (n=4). (ns, non-significant: ****$p < 0.0001$; ANOVA)

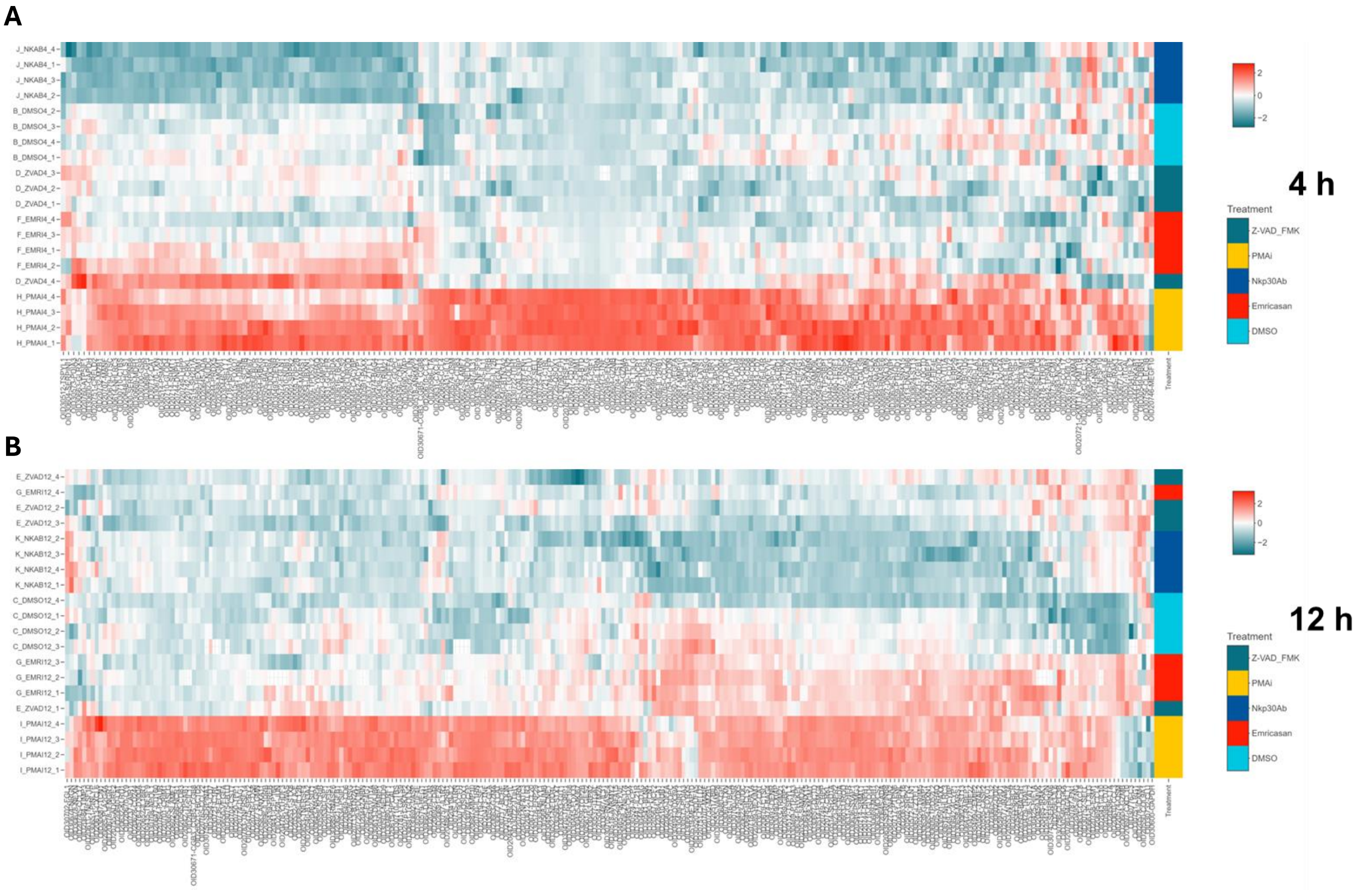

Supplementary figure 2. Full secretome analysis of pan-caspase inhibitor-treated NK cells.

NK lymphoma cell line KHYG-1 was incubated with DMSO (0.5 %; light blue), Z-VAD-FMK (50 µM; green), emricasan (50 µM; red), agonistic NKp30 antibody (1 µg/mL; dark blue), or PMAi (10 ng/mL PMA and 1 µg/mL ionomycin; yellow) for 4 h or 12 h (each condition n=4) after which supernatants were collected and relative protein levels were analyzed by PEA Olink panels Inflammation I and Inflammation II. Heatmaps of all secreted proteins with significantly different protein levels among the five treatment groups at 4 h **(A)** and 12 h **(B)**.
